# Geospatial Perspectives on the Intersection of Chronic Disease and COVID-19

**DOI:** 10.5888/pcd19.220145

**Published:** 2022-06-30

**Authors:** Jeremy Mennis, Kevin A. Matthews, Sara L. Huston

**Affiliations:** 1 Temple University, Philadelphia, Pennsylvania; 2 Office of the Associate Director for Policy and Strategy, Centers for Disease Control and Prevention, Atlanta, Georgia; 3 Muskie School of Public Service, University of Southern Maine, Portland, Maine; 4 Maine Center for Disease Control and Prevention, Augusta, Maine

This collection of articles in *Preventing Chronic Disease* (PCD) brings together scientists and practitioners from the breadth of public health and the social sciences to demonstrate how geospatial perspectives can contribute to understanding and addressing the intersection of chronic disease and COVID-19, a respiratory disease caused by the SARS-CoV-2 virus. The COVID-19 pandemic has affected chronic disease in many complex ways. Early in the pandemic, it became clear that people with chronic conditions and those in older age groups were at the highest risk for COVID-19 hospitalization and death (1–3). Racial and ethnic minority populations experienced disproportionately worse health outcomes (4). Pandemic-related disruptions to the health care system and individuals’ concerns about health care–related exposures affected chronic disease management: in-person visits for people with chronic conditions declined, supply chain disruptions led to shortages of medications, and the number of cancer screenings, treatments, and surgeries declined in the United States (5–7). More recent evidence suggests that COVID-19 may exacerbate existing chronic diseases and increase the risk of developing new chronic conditions, such as diabetes in adults (8,9), type 1 diabetes in children (10), neurological disorders (11), dementia (12), mental illness (13), and cardiovascular disease (14). In addition, an estimated one-half of COVID-19 survivors worldwide continue to have COVID-related health problems 6 months or more after recovery from the acute infection, making “long COVID” our newest and still largely unresearched chronic disease (15). Finally, social and economic inequities underlie disparities in incidence of both chronic diseases and COVID-19, an intersection that has been labeled a syndemic, defined as the “presence of 2 or more disease states that adversely interact with each other, negatively affecting the mutual course of each disease trajectory, enhancing vulnerability, and which are made more deleterious by experienced inequities” (16).

Space and place are key elements of individual and population health — social and environmental determinants of health are embedded within place, and health outcomes and inequities typically exhibit strong geographic variation (17,18). Thus, geospatial perspectives, which address aspects of space and place, play a key role in the public health response to the COVID-19 pandemic and its intersection with chronic disease (19,20). Here, we consider geospatial perspectives to include the broad swath of geospatial data, analytical techniques, and technologies encompassed in the field of geographic information science and technology (GIS&T) (21). Geospatial data on disease incidence and mortality, available at the individual address level or aggregated to small areas, allow us to understand the geographic distribution of COVID-19 and the chronic disease burden and their spatial coincidence with other measures. Geospatial data can also capture community-level socioeconomic characteristics, such as indicators of race, ethnicity, and class, which serve to illuminate interrelated disparities in the incidence of COVID-19 and chronic disease.

Geospatial analytical techniques support the investigation of ecological and individual-level associations among chronic diseases and COVID-19 outcomes. These techniques include mapping and computational and statistical methods adapted explicitly for spatial data analysis, such as geographically weighted regression. Incorporating geospatial data about environmental characteristics and human dynamics, such as local climate and human mobility patterns, can inform analyses of how individual and environmental characteristics interact to produce population-level outcomes of COVID-19 and chronic disease. Geospatial technologies, such as GPS (global positioning systems), satellite remote sensing, and geographic information systems (GIS) software, provide the technological infrastructure to collect and integrate these geospatial data, apply these geospatial analytical techniques, and publicly disseminate data and information through web-based mapping and geospatial data dashboards.

In this collection, the commentary by Smith and Mennis provides an overview of the role of GIS&T in responding to the COVID-19 pandemic, emphasizing the use of geospatial technologies for collecting data on disease prevalence, analyzing the spread of infection, communicating with the public, and optimizing the distribution of resources (22). The article is enlightening in depicting the use of GIS&T in the initial phase of the pandemic, when geospatial data and analyses were key to understanding the spread and transmission of the disease and the efficacy of nonpharmaceutical interventions, such as business closures and government directives that limited social gatherings.

Other contributions in this collection highlight how the authors used GIS&T to inform chronic disease and COVID-19–related policies, interventions, and public health communications. Foraker et al illustrate one such approach for leveraging GIS&T to support spatially directed interventions by developing a custom geospatial software application for visualizing the locations of COVID-19 cases at the individual residence level (23). This interactive mapping application can target public health responses to emerging disease hotspots and highlights the challenge to regional analyses of residential address-level data, which are typically restricted to authorized public health officials within a single jurisdiction. The research brief by Moise describes a spatial interpolation method that disaggregates zip code–level rates of COVID-19 to the census block group–level to facilitate the use of consistent, small-area spatial support when measuring associations with selected measures of social determinants of health (24).

Many of the contributions in this PCD collection focus on how GIS&T can be used to investigate the association between community attributes and health disparities, measures of social determinants of health, or risk factors related to chronic disease and COVID-19 outcomes. For example, the GIS Snapshots article by DuClos et al reports on a web browser–based software application that displays choropleth maps of chronic disease–related risk factors, hospitalizations, mortality, and the Economic Hardship Index at the county and zip code levels (25). This tool was designed to inform COVID-19 preparedness and response efforts at the local level by identifying communities particularly vulnerable to COVID-19. This map application provides an example of how state and local health departments work to provide access to substate-level data on chronic disease.

Two articles in this PCD collection examined whether the prevalence of a chronic disease geographically coincides with the prevalence of COVID-19. In research by Embury et al, subcounty data from San Diego County, California (which includes urban and rural areas), were used to explore whether spatial modeling of chronic disease rates and selected social determinants of health measures could identify communities most vulnerable to COVID-19 (26). The authors divided data on the pandemic into 5 time frames and examined how relationships between social determinants of health, chronic disease, and COVID-19 changed over time. Jansen et al tested whether the prevalence of respiratory illness was associated with COVID-19 mortality rates among older adults in Connecticut and Rhode Island (27). Educational attainment decreased the strength of the association, demonstrating that our understanding of COVID-19 outcomes can be improved by accounting for selected social determinants of health.

The pandemic’s impact on food supply and affordability, concurrent with rising unemployment and mobility restrictions, made food access difficult for many households. Lowery et al used mapping to illustrate how the closure of food stores accepting Supplemental Nutrition Assistance Program (SNAP) via electronic benefits transfer (EBT) during the pandemic reduced food access within walking distance in a community in San Diego, California, where food insecurity was prevalent before the pandemic (28). Alternatively, Beese et al showed that food access for SNAP participants in Washington State during the pandemic was enhanced by expanding food delivery services (29). Their maps showed that online food delivery services by grocery stores accepting SNAP via EBT increased substantially during the pandemic, enhancing food access for many low-income communities in the state. However, certain barriers to online delivery services, such as lack of broadband access, remain a challenge, particularly in rural areas.

Other research in this PCD collection focuses on the use of GIS&T to assess factors associated with the efficacy of pharmaceutical and nonpharmaceutical interventions to reduce transmission of SARS CoV-2 infection. In their GIS Snapshots article, Michaels et al found a significant positive correlation between household internet access and COVID-19 vaccination rates at the zip code–level in New York City and used bivariate choropleth mapping to display the areas most at risk of COVID-19 and those with the lowest levels of vaccination and internet access (30). When the analysis was conducted, many vaccine providers in New York City were offering only online systems to schedule appointments. The article highlights the importance of considering the digital health divide in addressing chronic disease, COVID-19, and health inequities.

Li et al leveraged a large, commercial geospatial data set of mobility data collected from GPS-enabled mobile phones in a national-level analysis of the association of COVID-19 outcomes with decreases in travel to common activity space locations, such as work and shopping (31). This research incorporated time lags in tests of association to investigate whether stay-at-home directives, business closures, and related policies that restricted mobility successfully reduced COVID-19 prevalence. Results showed a strong association between reductions in mobility to certain locations, such as workplaces, and declines in infection rates, particularly in urban areas, and demonstrated the efficacy of stay-at-home directives in the early stages of the pandemic.

This PCD collection demonstrates the diverse ways that GIS&T can support research and policy at the intersection of COVID-19 and chronic disease, including 1) the role of social and environmental determinants of health, 2) pharmaceutical and nonpharmaceutical interventions to mitigate the impact of the pandemic, and 3) data and information dissemination for public health practitioners and the public. However, we acknowledge certain limitations of the collection. These articles mainly focus on the early phases of the pandemic; many were written before the Delta and Omicron waves. Little attention was paid to areas of the US that, as of the time of this writing in May 2022, have had the highest rates of COVID-19 mortality, such as the Southeast, regions with high levels of social vulnerability, and rural areas (32). In addition, the collection does not consider the role of GIS&T in preventing or mitigating future waves of COVID-19 or the impact that COVID-19 may have on future geographic patterns of chronic disease. Furthermore, only one article in this collection was authored by practitioners at a public health agency (25), limiting our understanding of how geospatial perspectives on COVID-19 and chronic disease were deployed on the ground during the pandemic.

One important lesson of the COVID-19 pandemic is that the public health community cannot afford to continue regarding infectious disease and chronic disease as separate entities. Public health leaders have noted that “[a] challenge related to long-term COVID-19 sequelae is that we do not know yet the extent that COVID-19 exacerbates chronic disease, causes chronic disease, or will be determined a chronic disease unto itself” (33). Given the emerging evidence that COVID-19 not only exacerbates preexisting chronic disease but may also be a risk factor for developing heart disease, type 1 diabetes among children, depression, and other chronic diseases, geospatial approaches should be employed to identify areas of high rates of COVID-19 incidence that can be targeted for chronic disease surveillance, prevention, and the provision of health services.

We recognize that prevention and control of COVID-19 depend on prevention and management of chronic disease and vice versa, and the level of success in both depends on addressing the structural inequities in economic opportunity, racial and ethnic segregation, and resource accessibility that act as distal forces on more proximal social and environmental determinants of health, such as those associated with individual health behaviors. These structural mechanisms that affect health outcomes typically materialize in differences observed among places and regions. Geospatial approaches are thus critical for ecological analyses of disease incidence rates and for capturing and analyzing data on the structural social and environmental exposures that are key to understanding how COVID-19 and chronic disease intersect to produce individual health outcomes.

Another lesson from the COVID-19 pandemic is the need for interdisciplinary collaboration across the fields of public health, social science, and GIS&T and across teaching, research, and practice. Only 3 articles in this collection represent collaborations between epidemiologists or other public health professionals and geographers, who often serve as key GIS&T personnel in universities (22,26,31). We recommend that medical and public health investigators include GIS&T experts on their research teams, because they can enhance the translation of complex health findings by contributing to geospatial data acquisition and analytical plans adapted for geospatial data analysis during the earliest phases of research design. These experts can also identify geospatial data policies that may affect health studies, including the US Census Bureau’s 2020 Census differential privacy algorithm (34) or requirements for the maintenance of individual privacy in health research (35). GIS&T experts can also provide insights into how the axiomatic properties of spatial data (36) affect inferential statistical analyses, including violations of statistical independence (37), how the choice of geographic aggregation method can produce different results (the modifiable areal unit problem) (38), that statistical significance of coefficients often varies from place to place (spatial heterogeneity) (39), and the impact of data uncertainty on health studies (40–42).

The small number of collaborative articles in this collection also highlights the need for higher education to be an agent of change for building these collaborative networks. Academic public health programs can enhance the capacity for GIS&T in public health practice by partnering with the academic units within their institutions that already have GIS&T expertise. Graduates from such programs will be more employable in public health fields than graduates trained in GIS&T or public health alone. From a practitioner perspective, epidemiologists working in state and local health departments can benefit from continued and rigorous GIS&T training and resources, such as the Building GIS Capacity for Chronic Disease Surveillance program at the Centers for Disease Control and Prevention (43).

The most powerful contribution that GIS&T scientists can make in a rapidly changing public health environment is to use sound geospatial methods in the service of generating evidence-based public health policies. To ensure the choice of appropriate public health research questions and concordant analytical designs, GIS&T scientists should collaborate with public health researchers and be aware of how issues of health disparities and legacies of discrimination in health care (44) can affect geospatial health research designs and analyses. For example, nearly 23% of states reported that data on race and ethnicity were incomplete for COVID-19 cases in the early part of the pandemic, making it difficult to measure the pandemic’s impact on racial and minority populations (45). Qualitative geospatial techniques, such as those using georeferenced narrative data, can also play a central role in eliciting the lived experience of disenfranchised people and in examining how concentrated social and economic disadvantage, collective efficacy, exposure to violence, and other community-level characteristics shape individual health behaviors and outcomes that produce observed population-level health inequities (46).

Finally, we note that the pandemic has shed light on the need to strengthen national public health data infrastructures that support the integration of chronic and infectious disease data across various government agencies and facilitate public health data dissemination and communication for researchers, policy makers, and the public (47,48). This PCD collection highlights the critical need for incorporating a geospatial perspective into such efforts beyond the multiplicity of ad hoc mapping dashboards that have popped up over the past 2 years. Enhancing knowledge of cartography and interactive geospatial data visualization among software developers in the public health community is key to ensuring such tools are effective for science communication and assisting in public health intervention and prevention efforts.

In addition, eliminating barriers to the routine collection, geocoding, and sharing of residential address–level data in public health surveillance systems would provide more actionable data in this pandemic and the next. More broadly, the integration of geospatial perspectives into national public health data infrastructure initiatives to support future research on the intersection of chronic and infectious diseases can benefit from the experience of similar US government data infrastructure projects related to disaster and emergency response, where GIS&T plays a key role (49), such as The National Map (50), and the Disaster Risk Resilience Initiative (51). Data infrastructure development efforts should also be mindful of the confidentiality requirements for personal health information and incorporate recent developments in “geomasking,” which aims to preserve the anonymity of georeferenced observations, because location-based health data can potentially reveal personally identifiable information, even when aggregated over small areas (46). Some of these proposed efforts may present valuable opportunities for the new Center for Forecasting and Outbreak Analytics, launched by the Centers for Disease Control and Prevention in April 2022.

The public health community is only beginning to understand the profound and ongoing consequences of the interaction of chronic disease and COVID-19. This collection highlights the important role of GIS&T in understanding the social and environmental determinants of health that underlie inequities in infectious and chronic disease risk factors, ultimately producing the health disparities observed in outcomes from the intersection of COVID-19 and chronic disease. Interdisciplinary and collaborative efforts to expand geospatial perspectives in chronic disease prevention and treatment are crucial for responding to COVID-19 and future pandemics.
